# Niacin, an active form of vitamin B3, exerts antiviral function by recruiting β-arrestin through GPR109A to activate the phosphorylation of ERK and STAT1 axis

**DOI:** 10.1128/jvi.01519-25

**Published:** 2025-10-29

**Authors:** Shunran Li, Jin Zhao, Ziwen Song, Xinyu Zhang, Bing Lang, Congcong Wang, Huanle Luo, Jun Qian, Caijun Sun

**Affiliations:** 1 School of Public Health (Shenzhen), Sun Yat-sen University 626351 , Shenzhen, China; 2 Shenzhen Key Laboratory of Pathogenic Microbes and Biosafety, Shenzhen Campus of Sun Yat-sen University 582261 , Shenzhen, China; 3 Key Laboratory of Tropical Disease Control (Sun Yat-sen University), Ministry of Education, Guangzhou, China; 4 State Key Laboratory of Anti-Infective Drug Discovery and Development, School of Pharmaceutical Sciences, Sun Yat-sen University 626146 , Guangzhou, China; St Jude Children's Research Hospital, Memphis, Tennessee, USA

**Keywords:** niacin, vitamin B3, GPR109A, IFN-β, antiviral agent

## Abstract

The emergence of viral infectious diseases poses a significant threat to public health, and thus the development of effective and safe antiviral drugs is becoming an urgent priority in the pandemic era. Niacin is an active form of vitamin B3 and has been used for hyperlipidemia treatment for decades, with well-established safety and pharmacological profiles. In this study, niacin was found to be a promising antiviral agent with both nutritional and therapeutic benefits. Further data showed that niacin could bind to its receptor GPR109A and then recruit β-arrestin to promote the ERK-STAT phosphorylation axis, subsequently leading to the activation of interferon signaling. Therefore, these findings provided evidence for repurposing this well-established drug as an antiviral agent and also highlighted the potential of GPR109A as a novel target in antiviral therapy.

**IMPORTANCE:**

The frequent emergence of viral infections is a major concern for global health. Finding safe and effective antiviral treatments has become more urgent than ever. Niacin—a form of vitamin B3—has long been used to treat hyperlipidemia, and its safety is well-established in clinical applications. Notably, this research demonstrates niacin may also work as an antiviral agent, offering both nutritional and therapeutic benefits. Further studies show that niacin binds specifically to a receptor in host cells called GPR109A and then triggers a series of signals inside the cell that ultimately strengthen the host’s antiviral interferon system. These findings suggest that this widely available drug could be repurposed to fight against viruses, and that the GPR109A receptor might be a promising new target for antiviral drug development.

## INTRODUCTION

The frequent emergence of viral infectious diseases poses a significant threat to public health (1–3). Consequently, developing novel antiviral drugs is becoming a priority in the post-COVID-19 pandemic era. Recent studies have highlighted close interactions between antiviral immunity and various metabolic processes (4, 5). Notably, several key rate-limiting enzymes and metabolites in the tryptophan-kynurenine pathway play crucial roles in immune regulation and exhibit antiviral functions (6). We recently identified kynurenine-3-monooxygenase (KMO) and its metabolite quinolinic acid (QUIN) in the tryptophan-kynurenine pathway as having broad-spectrum antiviral activity (7). To further explore KMO-related metabolites with novel antiviral functions, we screened various structural analogs of QUIN to optimize its antiviral efficacy, safety, and pharmacokinetics.

Niacin, also termed nicotinic acid, or 3-pyridine carboxylic acid, is a structural analog of QUIN and widely recognized as a kind of nutrient that is an active vitamin B3. Niacin also serves as a precursor to NAD+ (nicotinamide adenine dinucleotide), which is essential to the plethora of cellular processes. Deficiency in niacin could lead to pellagra, with clinical symptoms such as dementia, diarrhea, and dermatitis, ultimately leading to death (8). Moreover, niacin has been extensively used as a medication for hyperlipidemia treatment for decades (9), with well-established safety and pharmacological profiles. However, the role of niacin in immune regulation and antiviral immunity remains poorly investigated.

In this study, we present the novel finding that niacin exerts antiviral functions and further elucidate the underlying mechanisms. Our findings demonstrate that niacin could be developed as a promising antiviral agent, combining both nutritional and therapeutic benefits. This study also enhanced our understanding of the complex relationship between cell metabolism and host antiviral immunity.

## MATERIALS AND METHODS

### Cell lines

293T cells (from the embryonic kidney of a female human fetus), Vero cells (from the kidney of a female normal adult African green monkey), A549 cells (from human alveolar adenocarcinoma basal epithelial cells), and Raw 264.7 cells (from macrophage of a male adult mouse) were cultured in complete Dulbecco’s modified Eagle’s medium (DMEM, Gibco) supplemented with 10% fetal bovine serum (FBS, Gibco) and 1% penicillin/streptomycin (Gibco), at 37°C in an atmosphere of 5% of CO_2_. The above cells were preserved in our laboratory.

A549 GPR109A knockdown cells were constructed in our laboratory. In brief, A549 cells were infected with sgRNA-expressing lentivirus with polybrene and then added with puromycin. The cells after puromycin screening were selected and validated by Western blotting analysis. The sgRNA sequences were listed in Table S1.

### Viruses

Vesicular stomatitis virus (VSV)-GFP was generously provided by Dr. Tian Lan (VectorBuilder, Guangzhou, China), while herpes simplex virus (HSV)-green fluorescent protein-luciferase is stored in our laboratory. These viruses were utilized in cell studies. The pathogenic HSV-1 McKrae strain, kindly gifted by Prof. Jumin Zhou (Kunming Institute of Zoology, Chinese Academy of Sciences, Kunming, China), was used for the animal study.

### Plasmid constructs

The full-length human GPR109A and β-arrestin1 and mouse GPR109A were cloned into the pcDNA3.1 vector, which is maintained in our laboratory and used as a mock transfection control in this study. The primers used for cloning were listed in Table S2.

### siRNA and cell transfection

Knockdown experiments were performed as previously described (10). In brief, cells at 80% confluence were transfected with various plasmids using Lipofectamine 2000 Transfection Reagent (Invitrogen), following the manufacturer’s instructions. After 5 h of transfection, the medium was replaced with DMEM containing 5% FBS and 1% penicillin/streptomycin, and the cells were incubated for an additional 24 or 48 h. siRNA oligonucleotide duplexes targeting GPR109A and β-arrestin were synthesized by Genepharma (Suzhou, China). According to the manufacturer’s protocol, cells were transfected with 100 nM of the indicated siRNAs using Lipofectamine RNAiMax Transfection Reagent (Invitrogen) for 48–72 h. The knockdown efficacy of the target genes was assessed by quantitative real-time PCR (RT-qPCR) or Western blot analysis. The sequences of all siRNAs are provided in Table S3, and the primers for RT-qPCR are listed in Table S4.

### RNA-seq library preparation, sequencing, and data processing

RNA-seq experiments were performed as previously described (11). In brief, Raw264.7 cells were incubated with or without niacin at a dose of 1 mM, after which the cells were infected with HSV-1 for 24 h, total RNA was extracted from the collected samples according to the manufacturer’s instructions, and RNA-seq libraries were generated using the TruSeq PE Cluster Kit v4-cBot-HS (Illumina, USA). The prepared libraries were sequenced on an Illumina platform by Sangon Biotech (Shanghai, China). Genes with *P*-values < 0.05 and |Log2 FC| > 1.5 were considered differentially expressed. Gene Ontology (GO) analysis was performed by using the GO knowledge base (https://geneontology.org/), and the Kyoto Encyclopedia of Genes and Genomes (KEGG) analysis was performed by using the KEGG database (https://www.kegg.jp/). The volcano plot was drawn by using the Volcano mapping tool in Hiplot (Tengyu Biotech, Shanghai, China).

### Luciferase assay

The Steady-Glo Renilla Luciferase detection system was utilized according to the manufacturer’s instructions (Promega, Madison, WI, USA).

### Mice

BALB/c mice were obtained from the Laboratory Animal Resource Center of Sun Yat-sen University and were bred in the SPF animal facility of Sun Yat-sen University in individually ventilated cages.

### *In vivo* therapy

BALB/c mice were bred in the SPF animal facility of the Laboratory Animal Resource Center of Sun Yat-sen University. Six- to 8-week-old mice were anesthetized, and corneal epithelial debridement was performed using a 30-gage needle, followed by the inoculation of 10^5^ plaque-forming units (PFU) HSV-1 (McKrae). Intraperitoneal injections of acyclovir (ACV) (5 mg/kg), niacin (5 mg/kg or 15mg/kg), or vehicle alone in 2% DMSO were administered daily for 1 week, and the ocular washes, disease scores, and corneal images (Carl Zeiss stereoscope) were acquired during the experiment. The corneal surface was washed with PBS (20 µL/eye) at various times post-infection, and then the virus titer of these samples was quantified by plaque assay.

### Challenge experiment in mice

BALB/c mice were infected with 1 × 10^5^ PFU HSV-1 (McKrae) by corneal inoculation as described above. The disease symptoms of experimental mice were monitored, and samples were collected as described above for subsequent analyzes.

### Enzyme-linked immunosorbent assay (ELISA)

The samples were collected and analyzed with the Mouse Interferon (IFN) Beta ELISA Kit (Solarbio Life Science, Beijing, China) according to the manufacturer’s instructions.

### ELISPOT

Enzyme-linked immunosorbent spot (ELISPOT) assays were performed as previously described (12). Briefly, 96-well plates (Millipore, Immobilon-P membrane) were coated with anti-IFN-γ monoclonal antibody (BD Pharmingen) overnight at 4°C and then blocked with 10% fetal bovine serum for 2 h at 37°C. Freshly isolated splenocytes were added in 4 × 10^5^ cells/well, and the HSV-1 peptides (Genscript, Nanjing, China) listed in Table S5 were immediately added at a final concentration of 2 mg/mL. The cells were incubated for 24 h at 37°C, and the expression of IFN-γ was then detected using biotinylated polyclonal anti-mouse IFN-γ (BD Pharmingen) and NBT/BCIP reagent (Pierce). Finally, the numbers of spots were quantified using an ELISpot reader (Bioreader4000, BIOSYS, Germany). The data were reported as spot-forming cells (SFC) per million cells.

### Western blotting analysis

The Western Blotting assay was performed as previously described (13). The antibodies used are listed in Table S6 in supplemental material.

### Cytotoxicity assay

A549, J2-BMM, and Vero cells, suspended in DMEM or RPMI 1640 with 10% fetal calf serum, were seeded into 96-well microtiter plates at a density of 1 × 10^4^ cells/well. Compounds to be screened were diluted with serum-free DMEM/ RPMI 1640 medium. These solutions were then added to the plates at various concentrations in a final volume of 100 µL. After 24 h, 10 µL of CCK8 reagent (Yeason, Shanghai) was added, and 4 h later, the value of OD450 was read by the microplate reader (Synergy HTX, Biotek). Compounds to be screened were listed in Table S7.

### Hematoxylin and eosin (HE) staining

Tissues from experimental mice were fully immersed in 4% paraformaldehyde, gradually dehydrated, embedded in paraffin, and cut into sections. HE staining was performed according to standard protocol by Wuhan Service Biotechnology CO, LTD.

### Statistical analysis

Statistical analyses were performed using GraphPad Prism software version 8 (GraphPad Software, Inc.). Statistical significance was calculated using Student’s two-tailed unpaired *t*-test or analysis of variance (ANOVA) with Holm-Sidak’s multiple comparisons test. *P < 0.05; **P < 0.01; ***P < 0.001.

## RESULTS

### Niacin is a potential antiviral agent

**Fig 1:**
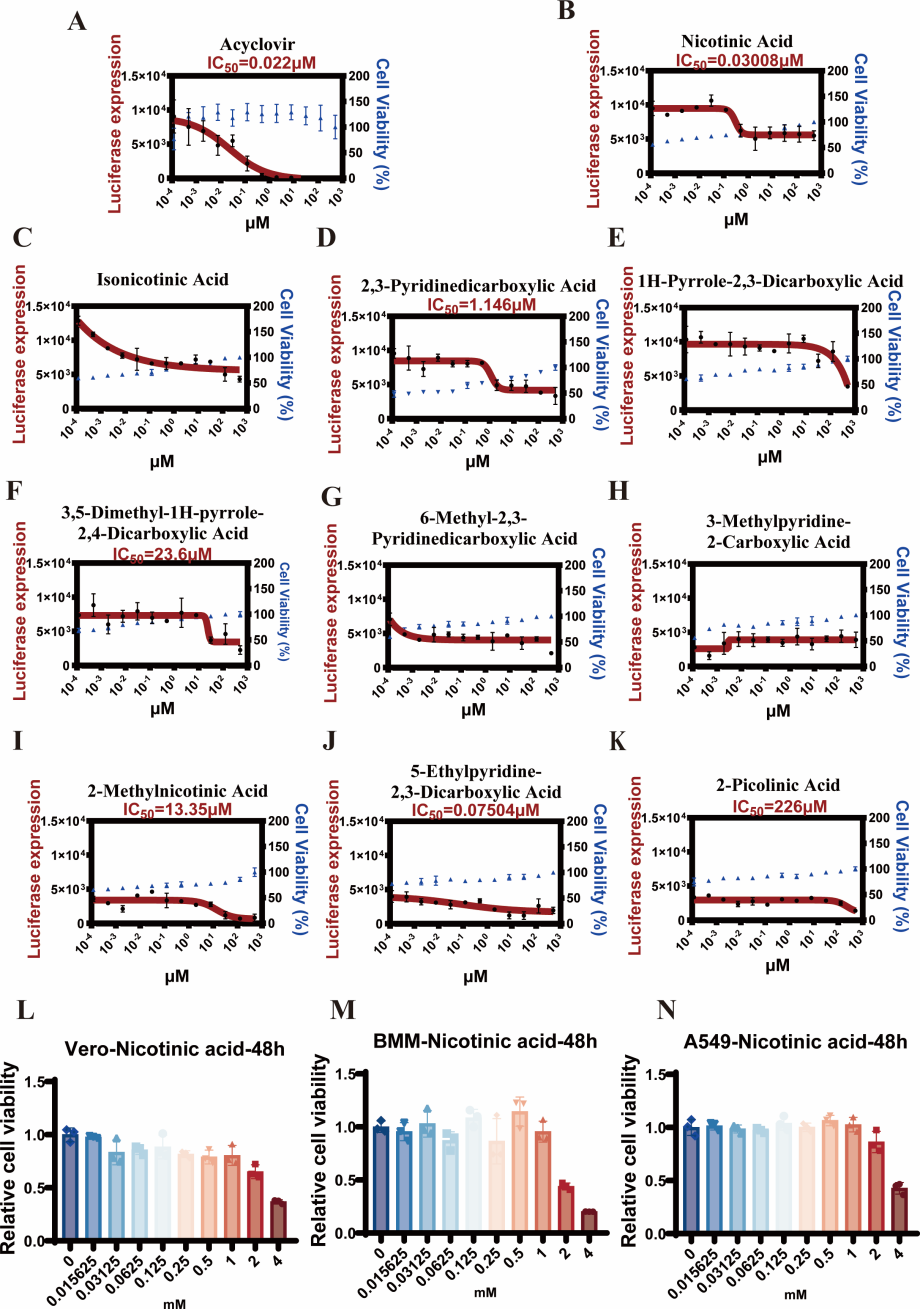
Niacin as a potential antiviral agent. (**A–K**) Vero cells were incubated with indicated compounds at indicated doses for 2 h, followed by infection with HSV-1 strains containing luciferase reporter gene (multiplicity of infection (MOI) = 0.1) for 24 h. HSV-1 expression was measured by luciferase assay. Cell viability was evaluated by CCK8 assay. (**L–N**) Cell lines were incubated with either niacin at the indicated doses for 48 h, and then CCK8 assays were performed to evaluate cell viability.

QUIN, also known as 2,3-pyridine carboxylic acid, has been identified in previous studies as a broad-spectrum antiviral agent (7). It is an essential metabolite in the tryptophan metabolism (14, 15). Structurally, it comprises a pyridine ring scaffold, with carboxyl groups connecting to the carbon atoms. To find more effective and safer antiviral drugs, we screened a series of compounds that bear structural similarity to QUIN (Fig. S1). To assess viral inhibition, HSV-1 containing a luciferase reporter gene (7) was used to infect Vero cells treated with the corresponding compounds. Subsequently, cell viability was assessed using the CCK-8 assay (Fig. 1A through K). Our results showed that niacin exhibited promising performance in these assays (Fig. 1B), so we further evaluated its cytotoxicity in Vero, BMM, and A549 cells (Fig. 1L through N). Our findings indicated that niacin as a pharmaceutical agent demonstrated both favorable safety profiles and effective antiviral properties.

### Niacin inhibits HSV-1 replication in various cell lines

**Fig 2:**
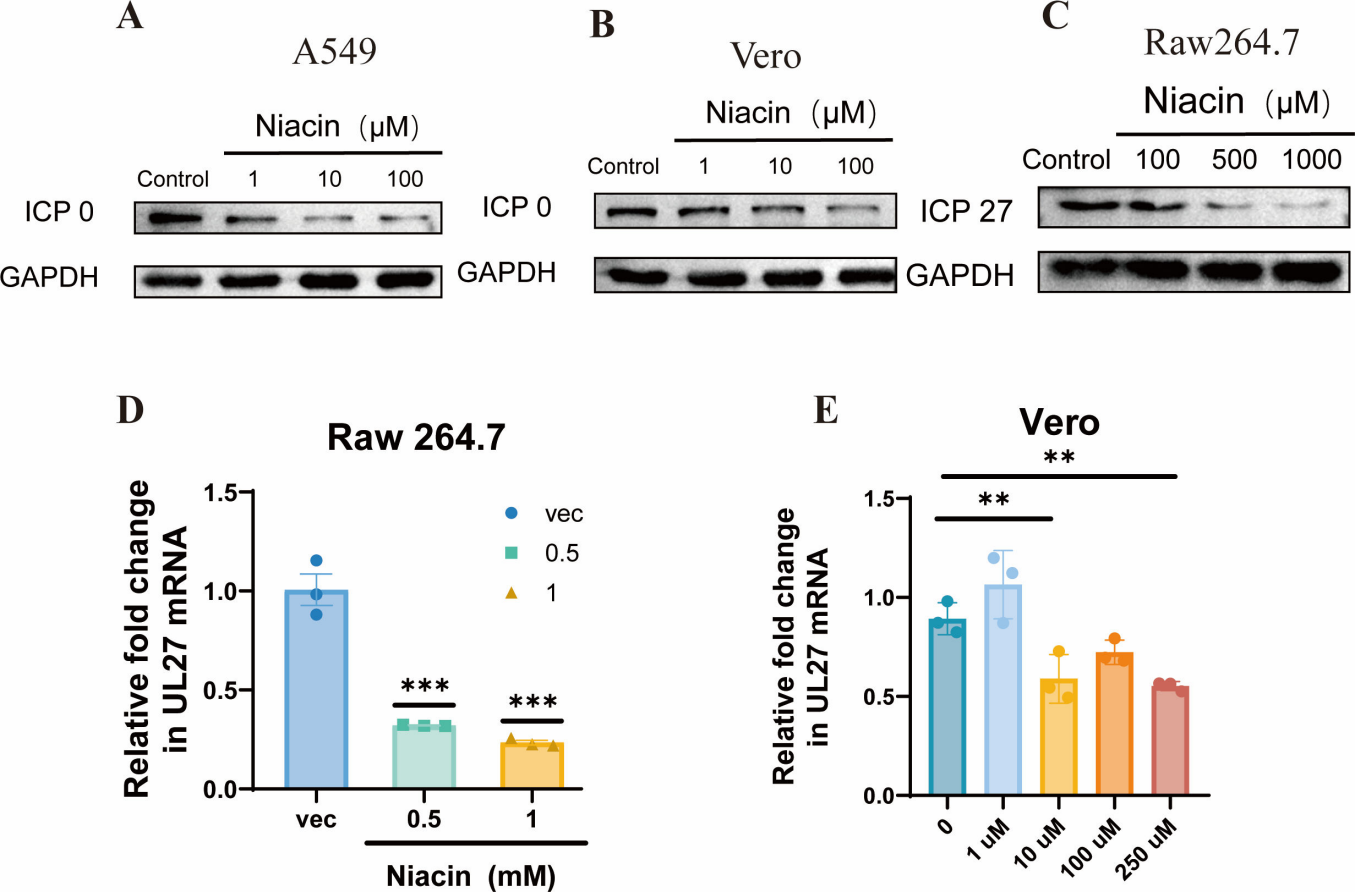
Niacin inhibits HSV-1 replication in different cell lines. Cell lines were treated with niacin at indicated doses while infected with HSV-1 (multiplicity of infection [MOI] = 0.1) for 24 h. (**A–C**) Cell lysis was used to measure the relative expression of HSV ICP0 or ICP27 by Western blot. (**D**) The expression level of HSV-1 UL27 was quantified by RT-qPCR. (**E**) Vero cells were incubated with 0, 1, 10, 100, and 250 µM niacin and HSV (MOI = 0.1) for 12 h, and then the expression level of HSV-1 UL27 was quantified by RT-qPCR. The data from at least triplicates were shown as the mean ± SD. *P < 0.05, **P < 0.01, ***P < 0.001.

To further verify the antiviral effect of niacin, experiments were performed to evaluate its activity across various cell lines. Niacin, at concentrations of 0, 1, 10, 100, 500, and 1,000 µM was co-incubated with the HSV-1 virus and the indicated cell lines for 24 h. Subsequently, the expression of HSV ICP0 or ICP27 was measured by Western blot, and results showed that niacin had the ability to inhibit HSV replication across multiple cell lines in a dose-dependent manner (Fig. 2A through C). Additionally, the anti-HSV-1 effect of niacin in Raw264.7 and Vero cells was confirmed by RT-qPCR (Fig. 2D through E) and plaque assay, respectively (Fig. S2). These findings collectively indicated the inhibitory effect of niacin against HSV-1.

### Antiviral efficacy of niacin in a highly pathogenic HSV-1 infection mouse model

**Fig 3:**
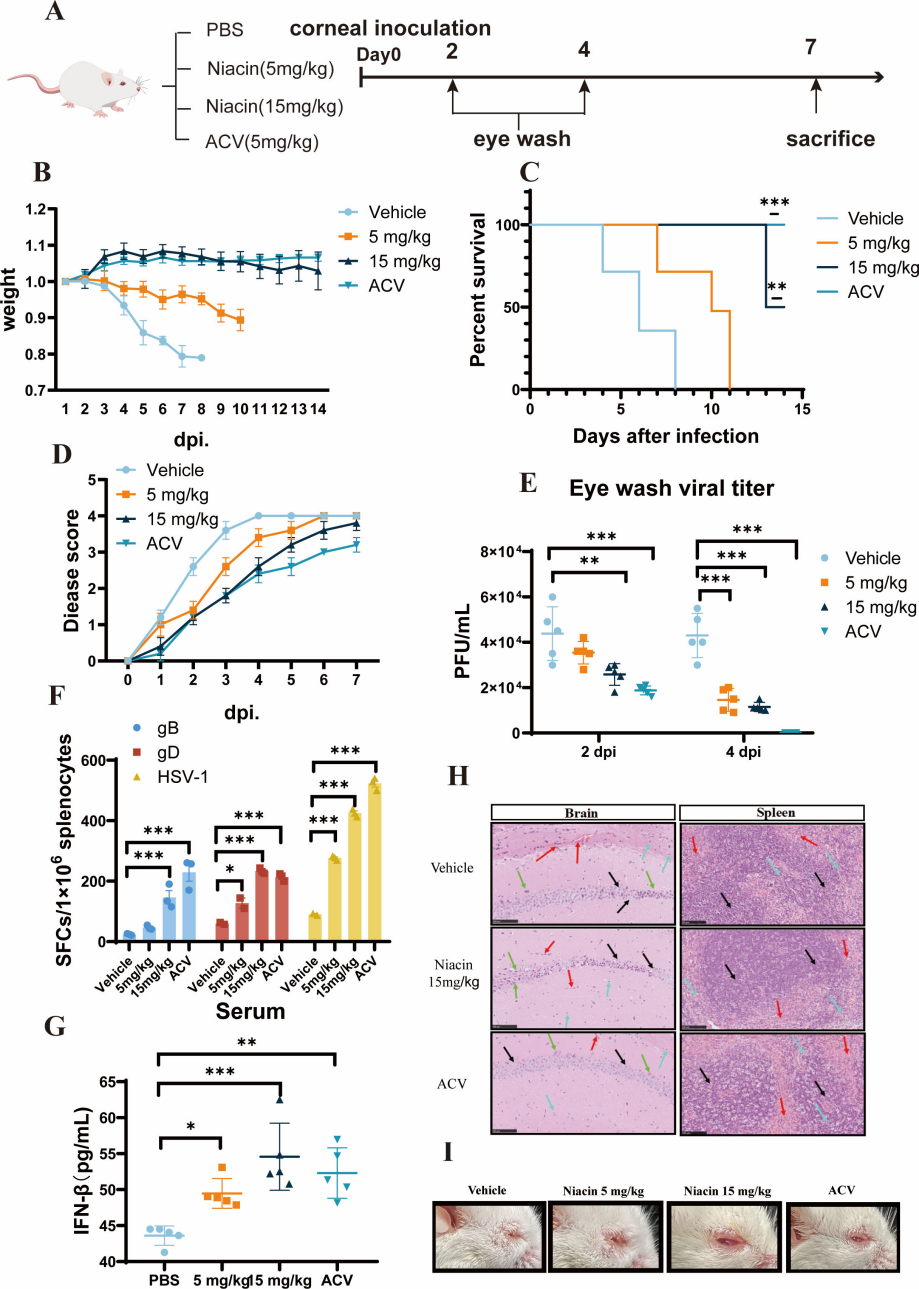
Antiviral efficacy of niacin in a highly pathogenic HSV-1 infection mouse model. The animal study was approved by the Institutional Review Boards and Animal Care and Use Committees of Sun Yat-Sen University (Approval No. SYSU-IACUC-2023-B029). All procedures were performed in strict accordance with relevant guidelines and regulations governing the ethical use of animals in research. (**A**) Schedule for evaluating the therapeutic efficacy of niacin in mice. ACV: Acyclovir. (**B**) Mice weight was measured each day for 14 days after the challenge. (**C**) Survival curve of experimental mice (*n* = 5). (**D**) Statistical analysis of disease scores of experimental mice (*n* = 5). (**E**) The HSV-1 titer in the eye washing fluid at 2 dpi and 4 dpi was measured by plaque assay (*n* = 5 per group). (**F**) The frequency of HSV-1 antigen-specific IFN-γ-secreting cells was determined by ELISPOT assay at 7 dpi. Data represented the spot-forming cells (SFC) per million cells. gB and gD refer to glycoprotein B and glycoprotein D, which are HSV surface glycoproteins that mediate viral entry into host cells. In this experiment, they were used as stimulating antigens to detect HSV-specific immune responses. (**G**) The concentration of IFN-β in the serum of experimental mice at 7 dpi was measured by ELISA assay (*n* = 5 per group). (**H**) The pathological changes in the experimental mice’s brain and spleen were observed by hematoxylin-eosin (HE) staining at 7 dpi. The scale bars: 100 µm. (**I**) The representative picture of progressive corneal scarring and visual impairment of experimental mice in different groups at 7 dpi. ACV: Acyclovir. dpi: days post-infection. The final data were presented as the mean ± SD of triplicate experiments. *P < 0.05, **P < 0.01, ***P < 0.001.

To investigate the antiviral efficacy of niacin *in vivo* and assess its potential as a therapeutic agent, we employed a murine model infected with a highly pathogenic strain of HSV-1 McKrae. Adult mice aged 6–8 weeks underwent an incision in their eyes, and HSV-1 McKrae was inoculated at a dose of 10^5^ PFU/mice. We administered treatments via intraperitoneal injection starting from Day 0, with daily doses thereafter. Acyclovir was utilized as a positive control to evaluate the protective effect of niacin against viral infection while PBS was used as a negative control (Fig. 3A). Our data demonstrated that niacin treatment significantly increased the weight and survival rate of HSV-infected mice (Fig. 3B and C). Additionally, disease scores were assigned to assess symptomatic improvement in infected mice following niacin administration (Fig. 3D). Scores ranged from 0 to 4. Mice exhibiting no signs of infection and maintaining normal activity receive a score of 0, while mice displaying symptoms such as trembling, huddling, evident ocular infection, and significant weight loss receive a score of 4.

On the 2nd and 4th day post-infection, eye wash samples were collected from the mice to measure viral titers. We found that niacin treatment reduced the viral titers in these mice eyewash samples (Fig. 3E). Subsequently, the mice were euthanized on the 7th day post-infection, and serum and splenic lymphocytes were harvested for ELISA and ELISpot assays. Our findings indicated that niacin therapy increased the frequency of HSV-1 antigen-specific IFN-γ-secreting cells (Fig. 3F) and elevated IFN-β levels in the plasma of HSV-infected mice (Fig. 3G). Niacin treatment also mitigated pathological damage in the brain and spleen of HSV-infected mice (Fig. 3H). In addition, niacin treatment reduced the progressive corneal scarring and visual impairment (Fig. 3I).

Furthermore, we assessed the prophylactic efficacy of niacin against HSV-1 infection by administering daily injections for 7 days prior to viral challenge. Mice were monitored for 15 days post-infection. Results demonstrated that pretreatment with niacin significantly enhanced survival rates and mitigated weight loss in infected mice (Fig. S3A and B), and reduced disease scores and viral titers at the primary infection site (Fig. S3C and D), achieving effects comparable to niacin treatment initiated concurrently with infection. However, its efficacy in suppressing viral replication within organs was weaker compared with the infection site (Fig. S3E), and no significant impact was detected on plasma IFN-β levels or the frequency of spleen-derived antigen-specific T cells (Fig. S3F and G). Therefore, niacin demonstrated significant therapeutic antiviral efficacy and moderate prophylactic protection.

### Niacin restricts virus replication via GPR109A

**Fig 4:**
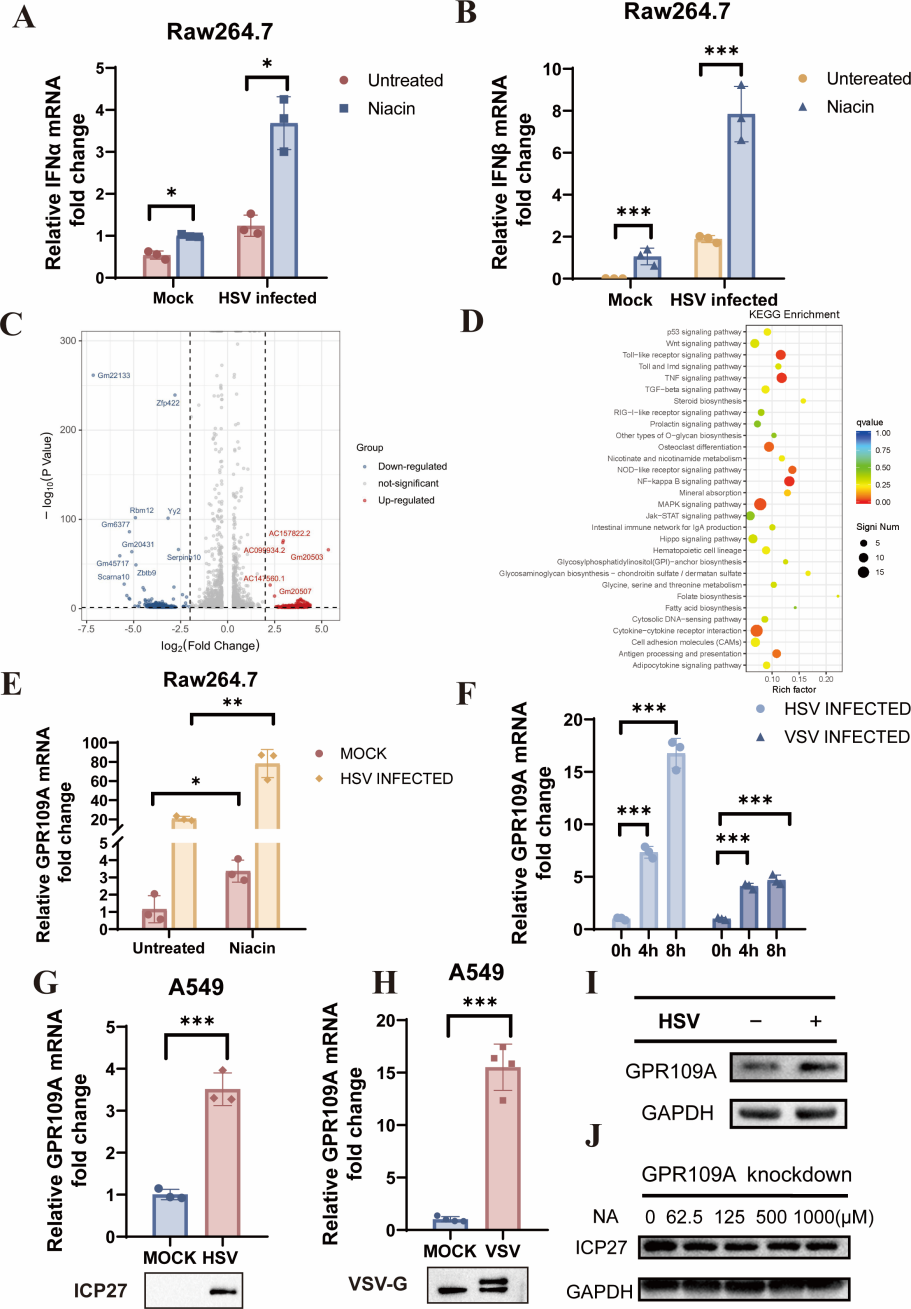
Niacin restricts virus replication via the upregulation of GPR109A. Raw264.7 cells were incubated with DMSO or 1 mM niacin for 12 h, with or without HSV-1 infection for 12 h (MOI = 0.1). Then cells were harvested, and total RNA was extracted. (**A and B**) The expression of IFN-α and IFN-β was measured by RT-PCR. (**C and D**) Analysis of transcriptome sequencing. The volcano graph was drawn by Hilplot. The right panel is the scatter plot of GO rich analysis. The color of the dots represents the Q value, and the size of the dots represents the *P*-value. A big reddish dot means the pathway is significantly enriched. (**E**) Raw264.7 cells were infected by either HSV-1 (MOI = 0.1) for 12 h. The expression of GPR109A was measured by RT-PCR. (**F**) Raw264.7 cells were infected by either HSV-1 or VSV (MOI = 0.1) for the indicated time. RT-PCR was applied to measure the expression of GPR109A. (**G and H**) A549 was infected by HSV-1 or VSV (MOI = 0.1) for 12 h. The expression of GPR109A was measured by RT-PCR. (**I**) A549 was infected by HSV-1 for 12 h. Western blot was applied to measure the expression of GPR109A protein. (**J**) GPR109A knockdown A549 cells were co-incubated with HSV-1 (MOI = 0.1) and niacin for 12 h. Total cell lysates were used to quantify HSV replication by Western blot, Niacin (NA). The final data were presented as the mean ± SD of triplicate experiments. *P < 0.05, **P < 0.01, ***P < 0.001.

Next, we further explored the antiviral mechanism of niacin. Niacin significantly enhanced the expression of type I IFN (Fig. 4A and B). Results of transcriptome sequencing revealed that there were 1,091 upregulated genes and 1,617 downregulated genes in niacin-treated cells (Fig. 4C). GO enrichment analysis indicated significant enrichment in the Toll-like receptor signaling pathway, TNF signaling pathway, NF-kappa B signaling pathway, MAPK signaling pathway, and G protein-coupled receptors signaling (Fig. 4D). Combined with the literature review, we found that GPR109A, a G protein-coupled receptor that could activate the MAPK pathway, is a high-affinity receptor of niacin (16–18).

Based on this observation, we hypothesized that GPR109A may play a role in the antiviral properties of niacin. Then, we treated Raw 264.7 cells with niacin for 12 h, followed by infection with HSV-1 for 12 h. The data demonstrated niacin treatment significantly upregulated the expression of GPR109A, especially a remarkably synergistic upregulation under HSV-1 infection status (Fig. 4E). Meanwhile, we observed that the expression of GPR109A was significantly increased in various cell lines during HSV-1 and VSV infections (Fig. 4F through I). We further knocked down GPR109A in A549 cells by the CRISPR-Cas9 system (Fig. S4) and found that the inhibition effect on HSV ICP27 expression by niacin was diminished in the GPR109A knockdown A549 cells (Fig. 4J), suggesting the involvement of GPR109A in niacin’s antiviral function.

Previous studies have focused on the role of niacin as a hypolipidemic drug and a vitamin. Physiologically, niacin acts as a precursor to NAD^+^, which supplies protons for a variety of oxidation-reduction reactions within the cell, making NAD^+^ essential for a wide range of metabolic functions. To clarify whether the antiviral function of niacin is also associated with its effects on NAD^+^ and mitochondria activity, we used nicotinamide (NAM), which is the intermediate product of niacin generating NAD^+^, to treat cells, and our data indicated that NAM does not have antiviral activity (Fig. S5A and B) or the ability to enhance IFN or inflammatory factor secretion (Fig. S5C and D).

### GPR109A inhibits viral replication through upregulating type I IFN

**Fig 5:**
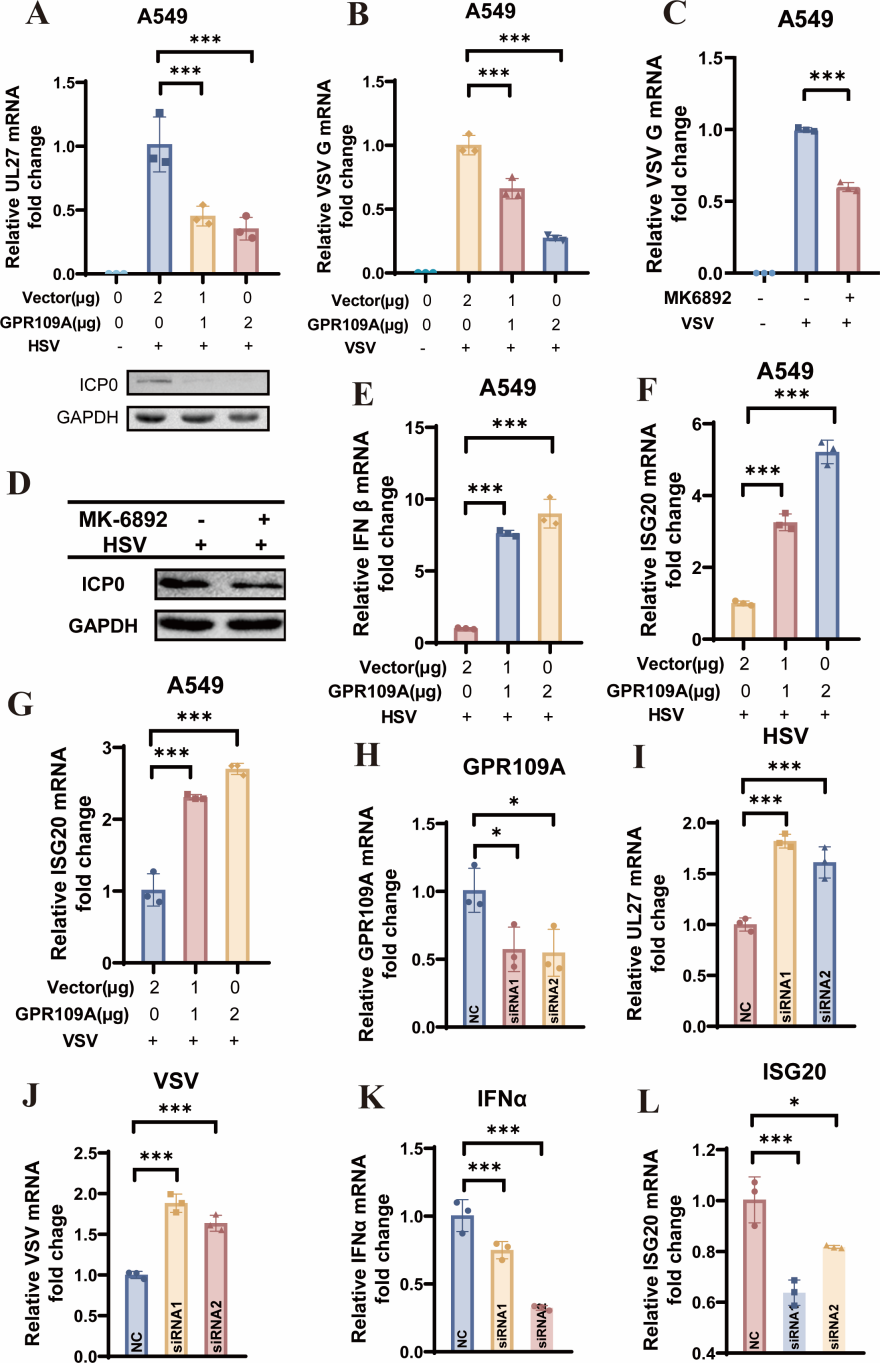
GPR109A inhibits viral replication through up-regulating type IFN. A549 cells were transfected with the indicated concentration of GPR109A plasmid for 24 h followed by HSV-1 or VSV infection for 12 h. (**A**) HSV UL27 expression was measured by RT-PCR, and ICP0 was measured by Western blot. (**B**) VSV G expression was measured by RT-PCR. (**C**) A549 cells were co-incubated with or without 1μM MK-6892 and VSV for 12 h. Then, VSV G expression was measured by RT-PCR. (**D**) A549 cells were incubated with or without 1μM MK-6892 while infected with HSV-1 for 12 h. The expression of HSV ICP0 protein was measured by Western blot. (**E–G**) A549 cells were transfected with the indicated concentration of pcDNA3.1-GPR109A plasmid for 24 h followed by HSV-1 or VSV infection for 12 h. pcDNA3.1-empty was used for vector control. Type I IFN and downstream ISG expression were measured by RT-PCR. (**H and I**) A549 cells were transfected with siRNA (targeting at position of 326 [siRNA1]/579 [siRNA2]) for 24 h followed by infection of HSV-1 for 12 h. Indicated gene expression was measured by RT-PCR. (**J**) A549 cells were transfected with siRNA (targeting at position 326 [siRNA1]/579 [siRNA2]) for 24 h followed by infection of VSV for 12 h. VSV G expression was measured. (**K and L**) A549 cells were transfected with siRNA (targeting at position 326 [siRNA1] / 579 [siRNA2]) for 24 h followed by infection of HSV-1 for 12 h. Indicated gene expression was measured by RT-PCR. Final data were presented as the mean ± SD of triplicate experiments. *P < 0.05, ***P < 0.001.

We then cloned the GPR109A gene into plasmid pcDNA3.1 and found that GPR109A overexpression could effectively inhibit HSV-1 and VSV replication by applying RT-PCR and Western blot (Fig. 5A and B). This inhibition effect in GPR109A overexpressing or enhancement effect in GPR109A knockdown cells was also confirmed using the TCID50 assay (Fig. S6A and B). Furthermore, MK-6892, a GPR109A agonist, also significantly suppressed viral replication (Fig. 5C and D). Consistent with niacin treatment, overexpression of GPR109A showed comparable results of upregulating type I IFN and IFN-stimulated genes (ISGs), mirroring the effects of niacin (Fig. 5E through G). To further elucidate the role of GPR109A, we designed siRNA to knock down GPR109A expression (Fig. 5H). Following knockdown, we observed the increased replication of HSV-1 and VSV (Fig. 5I and J), concomitant with decreased expression of type I IFN and its downstream ISGs (Fig. 5K and L). These findings indicated that GPR109A can exert its antiviral effects through upregulation of type I IFN expression.

### Antiviral effect of GPR109A relies on the β-arrestin-ERK-STAT1 axis

It is well known that G protein-coupled receptors (GPCRs) can react to diverse extracellular stimuli, and then transduce these signals to different cellular functional outputs mainly via two types of transducers, G proteins and arrestins (19–21).

**Fig 6:**
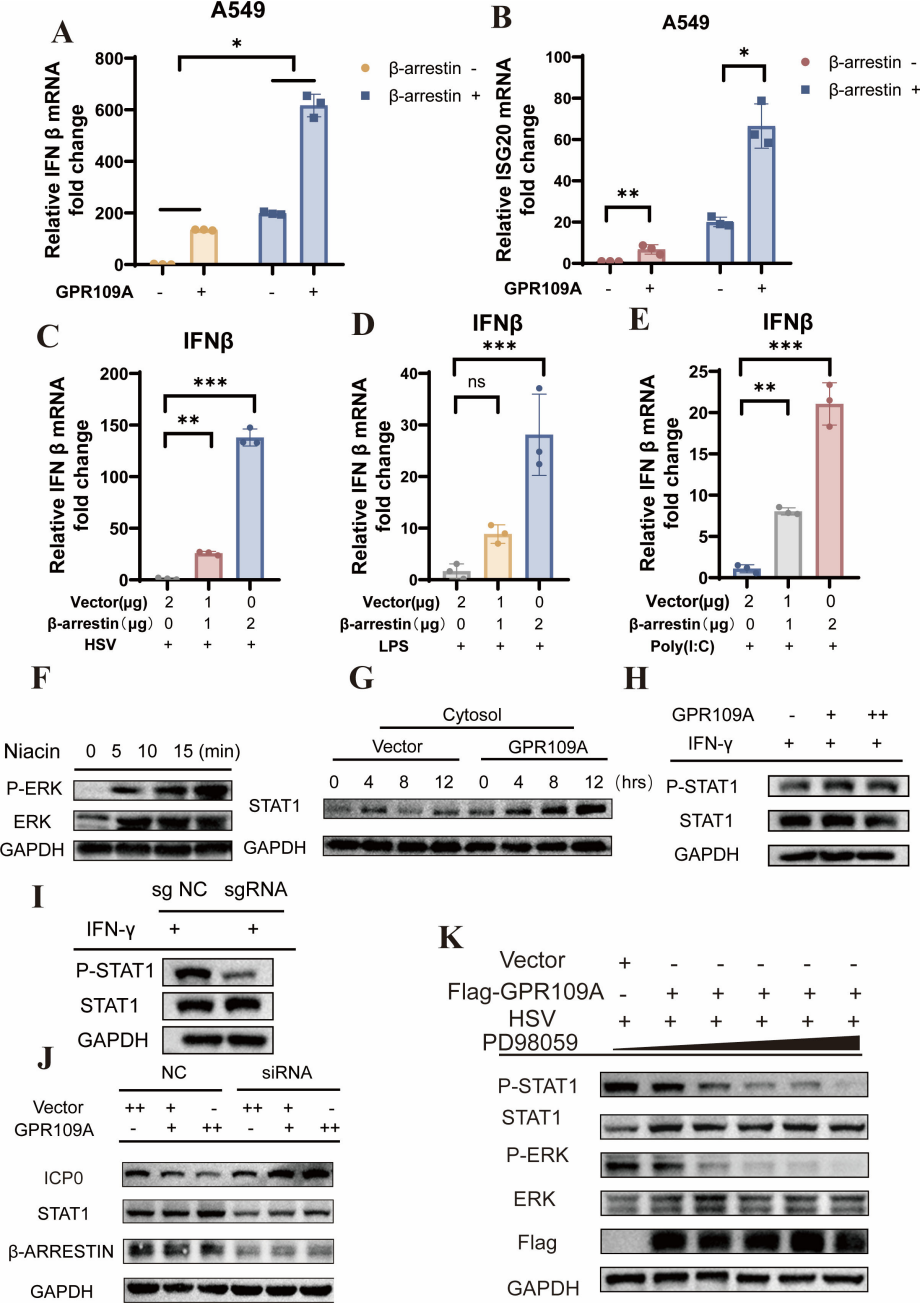
GPR109A can lead to the activation of IFN signaling through the arrestin-ERK-STAT1 axis. (**A and B**) A549 cells were transfected with pcDNA3.1-GPR109A or pcDNA3.1-β-arrestin1 plasmid for 24 h, followed by infection with HSV-1 (MOI = 0.1) for 12 h. RT-PCR analysis was used to quantify the transcription of IFN-β and ISG20. Vector: pcDNA3.1-empty. (**C–E**) A549 cells were transfected with indicated mass of pcDNA3.1-β-arrestin plasmid for 24 h, followed by stimulation by HSV-1 (MOI = 0.1), 10 µg/mL Poly(I:C), and 1 µg/mL LPS for 12 h. Total RNA was extracted for RT-PCR analysis. Vector: pcDNA3.1-empty. (**F**) 293T cells were transfected with pcDNA3.1-GPR109A plasmid for 24 h, then treated with 200 µM niacin for indicated time. Phosphorylation of ERK was measured by Western blot. (**G**) A549 cells were transfected with pcDNA3.1-GPR109A plasmid or vector for 24 h, followed by infection with HSV-1 for indicated time. Then the cytosol protein was extracted for quantifying the level of STAT1 expression by Western blot. Vector: pcDNA3.1-empty. (**H**) A549 cells were transfected with pcDNA3.1-GPR109A plasmid or vector for 24 h. Then cells were incubated with 20 ng/mL IFN-γ for 30 min. Cell lysis was used for Western blot. Vector: pcDNA3.1-empty. (**I**) A549 cells were infected with lentivirus containing negative control sgRNA or GPR109A sgRNA and then added with puromycin to screen. Then cells were incubated with 20 ng/mL IFN-γ for 30 min. Cell lysis was used for Western blot. (**J**) A549 cells were transfected with pcDNA3.1-GPR109A plasmid and siRNA targeting β-arrestin for 24 h followed by HSV-1 infection for 12 h. pcDNA3.1-empty plasmid and negative control RNA were used as negative control. Then, total cell lysis was used for Western blot assay. The expression level of HSV ICP0 protein, STAT1, β-arrestin, and GAPDH was measured. NC: negative control. (**K**) pcDNA3.1-GPR109A and vector plasmid were transfected in A549 cells for 24 h, and then PD98059 at a dose of 0, 5, 10, 15, 25, 50 µM and HSV-1 were co-incubated with cells for 12 h. Total cell lysis was used for Western blot assay. Final data were presented as the mean ± SD of triplicate experiments. ns: no significance. *P < 0.05, **P < 0.01, ***P < 0.001.

A previous study showed that β-arrestin1 can serve as an intracellular adaptor protein for GPR109A, mediating its downstream signaling (22). Our study showed that overexpression of β-arrestin1 also exhibited antiviral effects and upregulated type I IFN and downstream ISGs, similar to GPR109A (Fig. S7A and B). RT-PCR analysis of A549 cells co-transfected with GPR109A and β-arrestin1, followed by HSV-1 infection, revealed a synergistic interaction between these two proteins in upregulating IFN and downstream ISGs (Fig. 6A and B). Furthermore, β-arrestin1 promoted the secretion of type I IFN in response to the stimulation by HSV-1, LPS, Poly(I:C) (Fig. 6C through E) and downstream ISGs (Fig. S7C through F).

Since studies have reported that GPR109A can recruit β-arrestin1 to the cell membrane and promote the phosphorylation of extracellular regulated protein kinases (ERK) (22, 23), we therefore performed experiments to detect whether niacin treatment can affect the phosphorylation of ERK. As expected, niacin effectively promoted the level of ERK phosphorylation in GPR109A-transfected 293T cells (Fig. 6F). We further validated this effect using wild-type and GPR109A knockdown A549 cells treated with niacin (Fig. S8A and B). Additionally, knockdown of β-arrestin diminished niacin-induced ERK phosphorylation (Fig. S8C). Additionally, we found that GPR109A overexpression increased STAT1 expression (Fig. 6G), and inhibition of β-arrestin1 reduced the expression of STAT1 and enhanced the replication of HSV-1 (Fig. 6J). GPR109A also enhanced STAT1 phosphorylation induced by IFN γ (Fig. 6H), which was significantly reduced in GPR109A knockdown cells (Fig. 6I). Moreover, to clarify whether the phenomenon of increased STAT1 phosphorylation is mediated by ERK phosphorylation, we treated GPR109A-overexpressing cells with the ERK inhibitor PD98059, followed by HSV infection for 12 h, and we found that inhibition of ERK phosphorylation resulted in a significant reduction in STAT1 phosphorylation (Fig. 6K). Together, these findings demonstrated that GPR109A can recruit β-arrestin to promote the phosphorylation of ERK and STAT1, subsequently leading to the activation of IFN signaling.

To further investigate how GPR109A exerts its antiviral function, we constructed mutant variants of GPR109A. It is reported that the phosphorylation pattern of GPCRs might mediate the receptor endocytosis or its downstream signaling (24). Specifically, there is a predicted phosphorylation site S328 in the intracellular domain of GPR109A. The residue S328 to alanine or aspartate was mutated to assess the impact of GPR109A phosphorylation on antiviral and IFN-upregulating functions. Overexpression of corresponding plasmids in A549 cells revealed that the level of phosphorylation at residue S328 mitigated the antiviral function of GPR109A (Fig. S9A and B).

To understand the role of different structural domains of GPR109A in its antiviral function, we generated truncation mutants lacking the extracellular domain, the third and fourth transmembrane domains, or specific intracellular segments. Our results indicated that the extracellular, transmembrane, and intracellular domains were all crucial for the antiviral and IFN-upregulating functions of GPR109A (Fig. S9A and B). However, given that the structural integrity of GPCRs is crucial for their function of signal transduction, studies utilizing truncated forms may be interpreted with caution. To further elucidate the role of GPR109A signaling in antiviral activity, we compared multiple agonists with distinct signaling biases (Fig. S9C). Among these, our data showed that niacin exhibited the most potent antiviral effect. MK-6892 is a potent dual-pathway agonist which activates both Gi and β-arrestin pathways but demonstrates higher efficacy toward Gi protein activation. MK-0354 is a Gi-biased agonist with minimal β-arrestin recruitment . Our results demonstrate that agonists with stronger β-arrestin recruitment induce more potent antiviral effects, supporting our proposed mechanism that GPR109A-mediated antiviral activity depends on β-arrestin recruitment and subsequent ERK-STAT1 phosphorylation cascade.

## DISCUSSION

**Fig 7:**
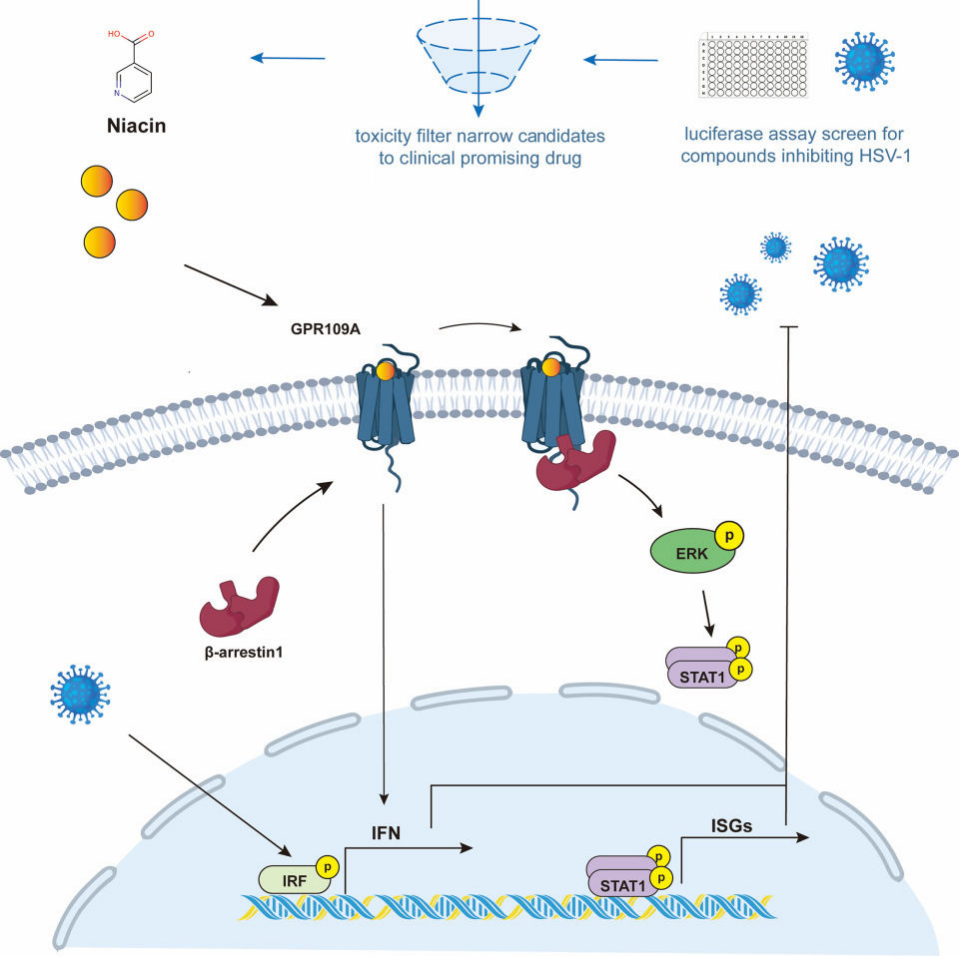
Schematic illustration of antiviral function of niacin and underlying mechanism. HSV-1 containing a luciferase reporter gene was employed to screen potential antiviral agents among QUIN analogs, and then the safety of compounds that were effective in inhibiting virus replication was evaluated by cell cytotoxicity assay. Niacin, an active form of vitamin B3, was found to be a promising antiviral drug candidate. Mechanically, niacin could bind to its receptor GPR109A and then recruit β-arrestin1 to promote the phosphorylation of ERK and STAT1 axis, subsequently leading to the activation of IFN signaling and enhancement of the expression of IFN-stimulating genes (ISGs).

In the present study, we reported that niacin exerts a novel antiviral function in a highly pathogenic HSV-1 infection mouse model. RNA-seq analysis and literature review (25–29) gave hints that the antiviral function of niacin was likely mediated by its high-affinity receptor GPR109A. Our evidence confirmed that GPR109A overexpression suppressed the replication of HSV-1 and VSV, upregulated Type I IFN and downstream ISGs. Additionally, GPR109A agonists besides niacin also exhibited antiviral effects. These findings suggested that GPR109A could be a potential target for antiviral drug development. Our further research demonstrated that the activation of GPR109A can upregulate ISG expression via the β-arrestin-ERK-STAT axis, thereby exerting antiviral effects (Fig. 7).

The development of antiviral drugs currently follows two main strategies: targeting virus cellular machinery or targeting host cells/cellular mechanisms involved in antiviral responses (30). Antiviral drugs approved by the FDA are primarily based on the first strategy, which provides good specificity and antiviral efficacy (30, 31). However, they often lack broad-spectrum antiviral function and are prone to resistance due to viral mutations under drug selection pressure. The host-targeting approach aims to enhance the host’s antiviral immunity, with IFNs being a promising target due to their broad-spectrum activity. However, host-targeting drugs often face challenges in drug ability and potential toxicity. As a kind of vitamin, niacin has a high safety threshold and has been used as an anti-hyperlipidemia drug for many years. Its pharmacokinetics are well-characterized. Our study demonstrated that niacin inhibited viral infection through interaction with its receptor GPR109A, providing valuable hints for expanding its indications of niacin as an antiviral drug candidate. More importantly, GPR109A is a member of the GPCR protein family. This suggests that by designing ligands for GPR109A, it may be possible to develop antiviral drugs with fewer side effects and better efficiency.

The therapeutic potential of niacin against viral infections remains poorly studied. While a Canadian clinical trial evaluated adjunctive niacin therapy in HIV infection (32), aiming to counteract tryptophan depletion and immune hyperactivation caused by HIV infection and thus improve CD4^+^ T-cell recovery, results showed that niacin significantly reduced plasma kynurenine levels and modestly decreased CD4^+^ T-cell activation (33). Critically, this approach utilized niacin primarily as a tryptophan supplement to enhance antiretroviral outcomes, not to probe its intrinsic antiviral properties. In addition, a screen of 1,500 compounds identified the niacin analog 6-aminonicotinamide (6-AN) as an inhibitor of hepatitis B virus replication (34). Notably, although 6-AN is structurally related to niacin, this study did not address niacin’s antiviral potential. Here, we report the first evidence that niacin exhibits direct and intrinsic antiviral activity by inhibiting HSV-1 replication.

Studies have also indicated the involvement of GPR109A in inflammation and tumorigenesis (27, 35, 36). GPR109A is highly expressed not only in white and brown adipose tissues but also in the spleen and various immune cells including monocytes, macrophages, dendritic cells, and neutrophils (26, 28, 29, 37, 38), but its role in antiviral immunity remains poorly understood. Our study demonstrates that activation of GPR109A suppresses HSV-1 replication by upregulating type I IFN and ISGs, thereby uncovering a previously unrecognized function of this receptor in innate antiviral defense. Given that GPR109A signaling can exhibit biased agonism, future studies identifying the specific adaptor proteins responsible for its antiviral effects and quantifying their contributions will be essential for developing targeted therapeutic strategies.

This study has several limitations. First, the antiviral mechanism mediated by the GPR109A–β-arrestin–ERK–STAT1 signaling axis, although clearly supported by our *in vitro* data, requires further validation using appropriate *in vivo* models. Second, the rapid disease progression in our highly pathogenic HSV-1 murine infection model might limit the ability to fully evaluate the long-term therapeutic efficacy of niacin. Animal infection model with extended disease courses would be more suitable for long-term assessment of post-exposure therapeutic regimens in future study. Third, while we demonstrated efficacy against HSV-1, the antiviral activity of nicotinic acid and GPR109A engagement against other viruses remains unexplored. Further studies assessing the preventive or therapeutic potential of niacin against diverse viral pathogens would significantly advance its potential clinical application as a broad-spectrum antiviral drug.

In summary, our study identified niacin can exert antiviral function through its receptor GPR109A-recruiting β-arrestin to promote the phosphorylation of ERK and STAT1 axis. Our findings provided evidence for repurposing this well-established drug as an antiviral agent and also highlighted the potential of GPR109A as a novel target in antiviral therapy.

## Data Availability

The data supporting the differential gene expression analysis are available in the supplemental material.

## SUPPLEMENTAL MATERIAL

Supplemental figures and tables jvi.01519-25-s0001.docx Figures S1 to S9 and Tables S1 to S7.

Differential gene expression analysis jvi.01519-25-s0002.xlsx Raw264.7 cells were incubated with or without niacin at a dose of 1 mM, after which the cells were infected with HSV-1 for 24 h, and total RNA was extracted from the collected samples to conduct RNA-seq, on which differential gene analysis is based.

ASM does not own the copyrights to Supplemental Material that may be linked to, or accessed through, an article. The authors have granted ASM a non-exclusive, world-wide license to publish the Supplemental Material files. Please contact the corresponding author directly for reuse.
