# Supplementary material for: Niacin, an active form of vitamin B3, exerts antiviral function by recruiting β-arrestin through GPR109A to activate the phosphorylation of ERK and STAT1 axis
Source: J Virol. 2025 Oct 29;99(11):e01519-25. doi: 10.1128/jvi.01519-25 (PMC12645984; doi:10.1128/jvi.01519-25)
Supplement: Supplemental figures and tables — Figures S1 to S9 and Tables S1 to S7. [file jvi.01519-25-s0001.docx]

Figure S1.


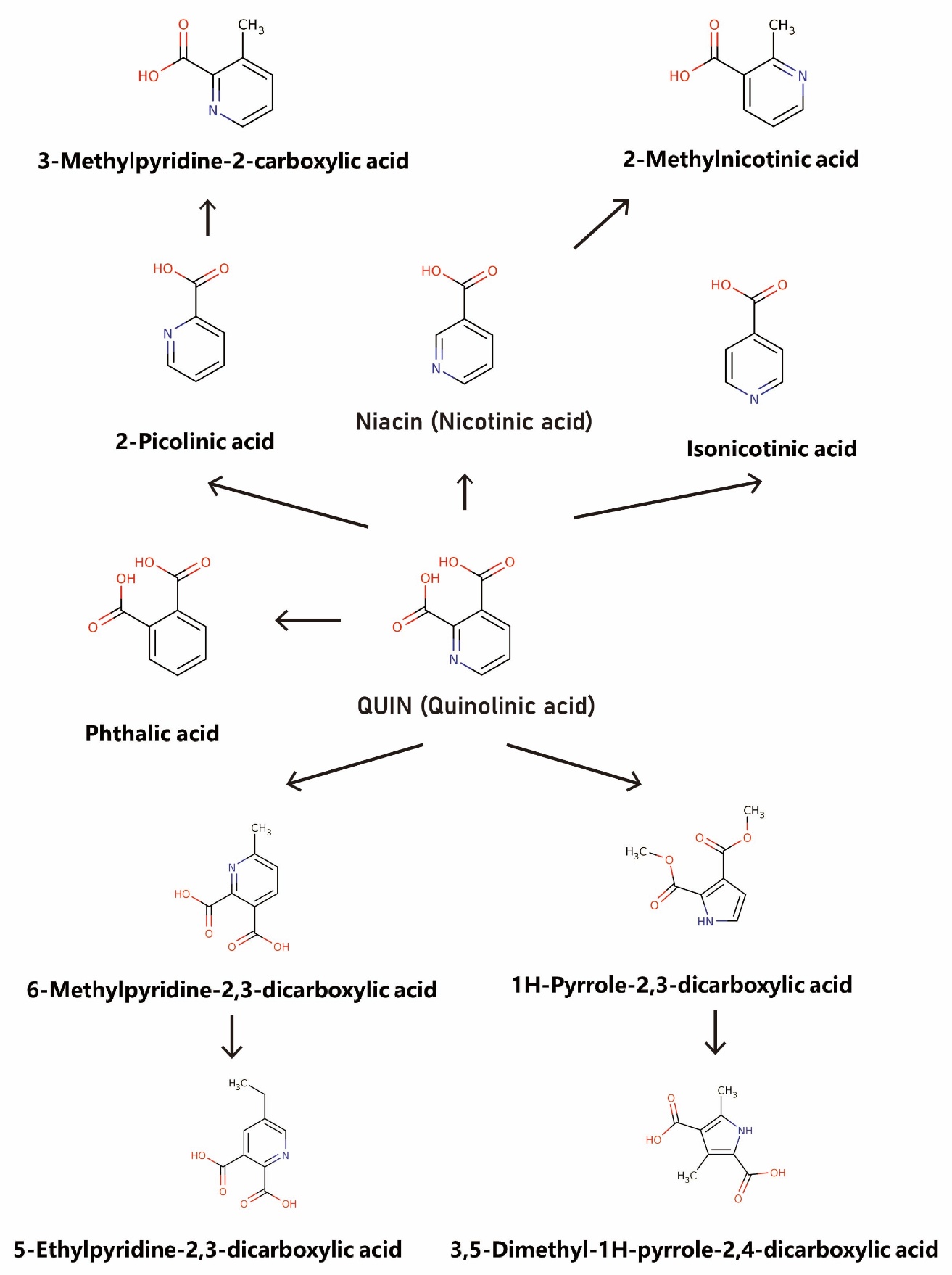


**Figure S1. Structural Formula of compounds screened in this study.**

Figure S2.


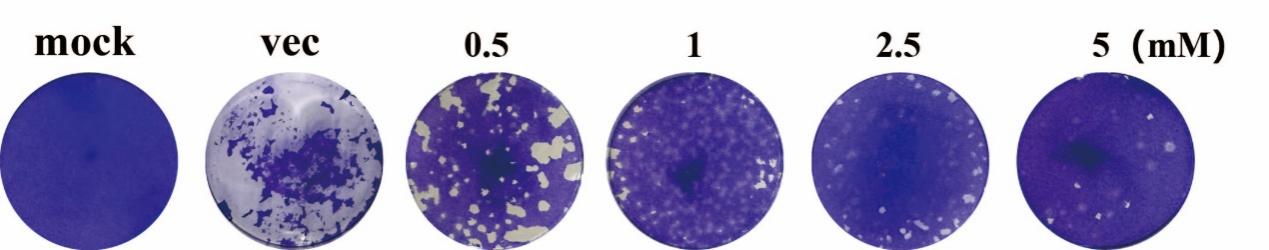


**Figure S2. Evaluation of the antiviral function of niacin by plaque assay.** Vero cells were treated with niacin at indicated doses while infected with HSV-1(MOI = 0.1) for 24h, and then the viral titer of the supernatant was measured by plaque assay.

Figure S3.


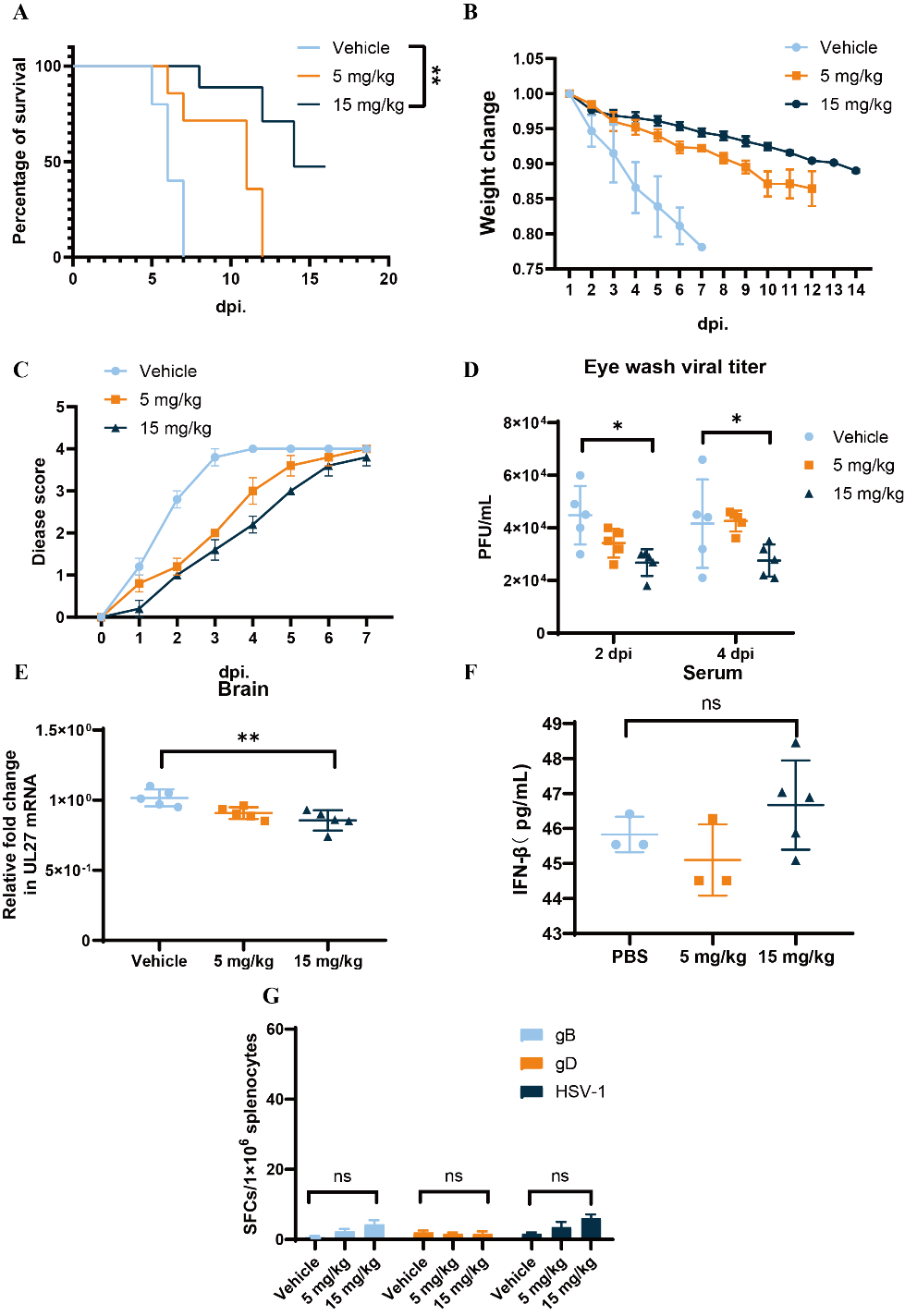


**Figure S3. Preventive efficacy of niacin in a highly pathogenic HSV-1 infection mouse model.** Mice were intraperitoneally injected daily with either PBS, 5 mg/kg or 15 mg/kg niacin for 7 consecutive days, followed by viral challenge. (A) Survival curve of experimental mice (n = 5) (B) Mice weight was measured each day for 14 days after the challenge. (C) Statistical analysis of disease scores of experimental mice (n = 5). (D) The HSV-1 titer in the eye washing fluid at 2 dpi and 4 dpi were measured by plaque assay (n =5 per group). (E) The relative quantity of HSV-1 UL27 mRNA in mouse brain tissues measured by RT-PCR at 7 dpi. (F) The concentration of IFN-β in the serum of experimental mice at 7 dpi was measured by ELISA assay (n =5 per group). (G) The frequency of HSV-1 antigen-specific IFN-γ-secreting cells was determined by ELISPOT assay at 7 dpi. Data represented the spot-forming cells (SFC) per million cells. The final data were presented as the mean ± SD of triplicate experiments. *P< 0.05, **P< 0.01.

Figure S4.


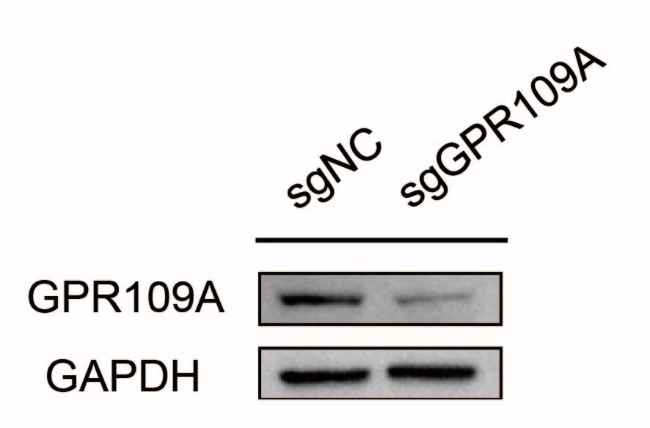


**Figure S4. Confirmation of GPR109A knock-down A549 cells.** The level of GPR109A expression in sgNC and GPR109A knock-down cells were detected by western blot assay.

Figure S5.


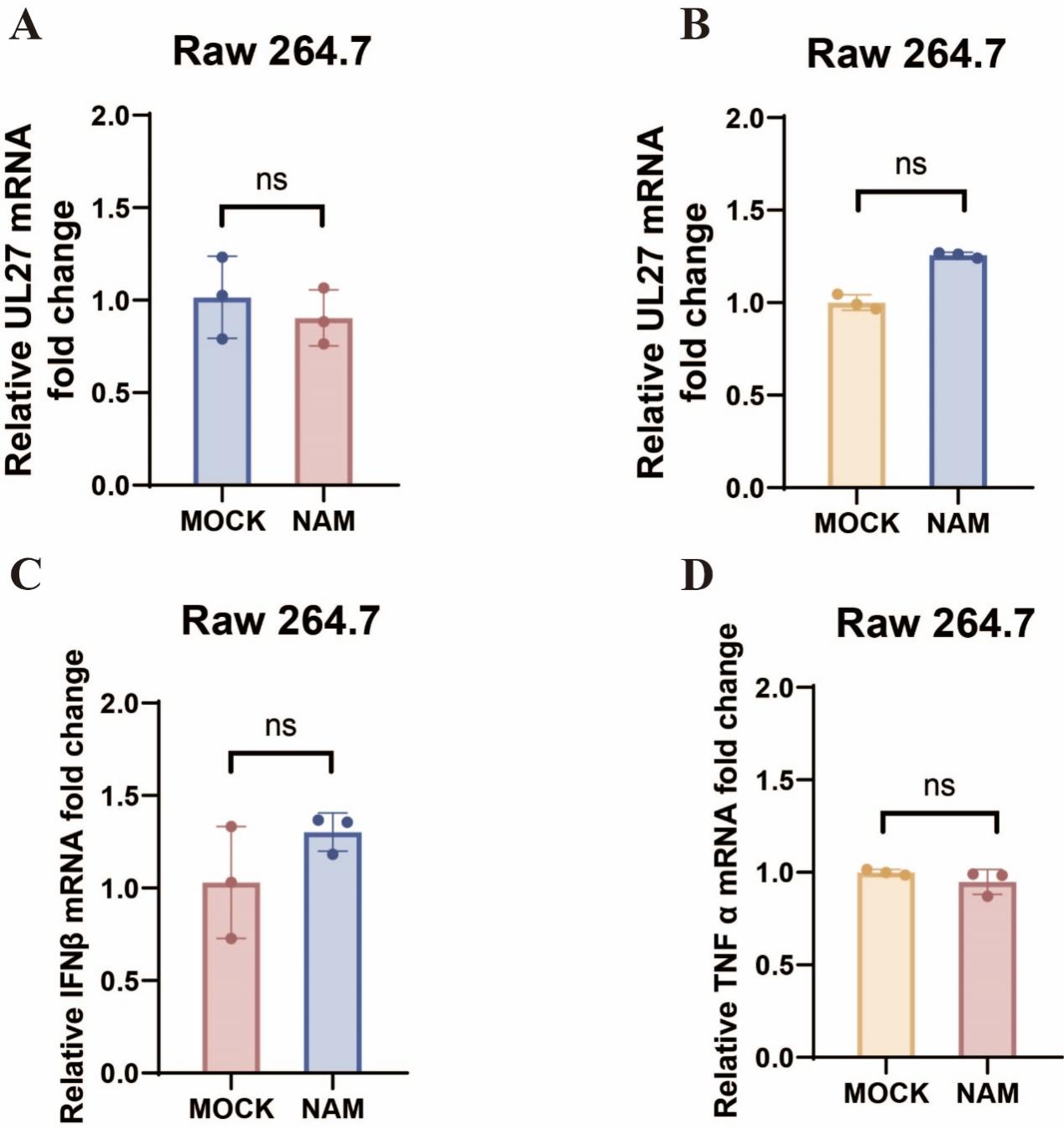


**Figure S5. Nicotinamide did not exert antiviral function.** Raw264.7 cells were treated with 1mM NAM while infected by HSV for 12h, and then total RNA was extracted for RT-PCR analysis. (A) The expression level of HSV UL27 in NAM or Mock treated Raw 264.7 cells infected by HSV-1(MOI=0.1). (B) The expression level of HSV UL27 in NAM or Mock treated Raw 264.7 cells infected by HSV-1(MOI=1). (C) The expression level of IFN-β in NAM or Mock treated Raw 264.7 cells infected by HSV-1(MOI=1). (D) The expression level of TNF-α in NAM or Mock treated Raw 264.7 cells infected by HSV-1(MOI=1). NAM: nicotinamide. Final data were presented as the mean ± SD of triplicate experiments. ns: no significance.

Figure S6.


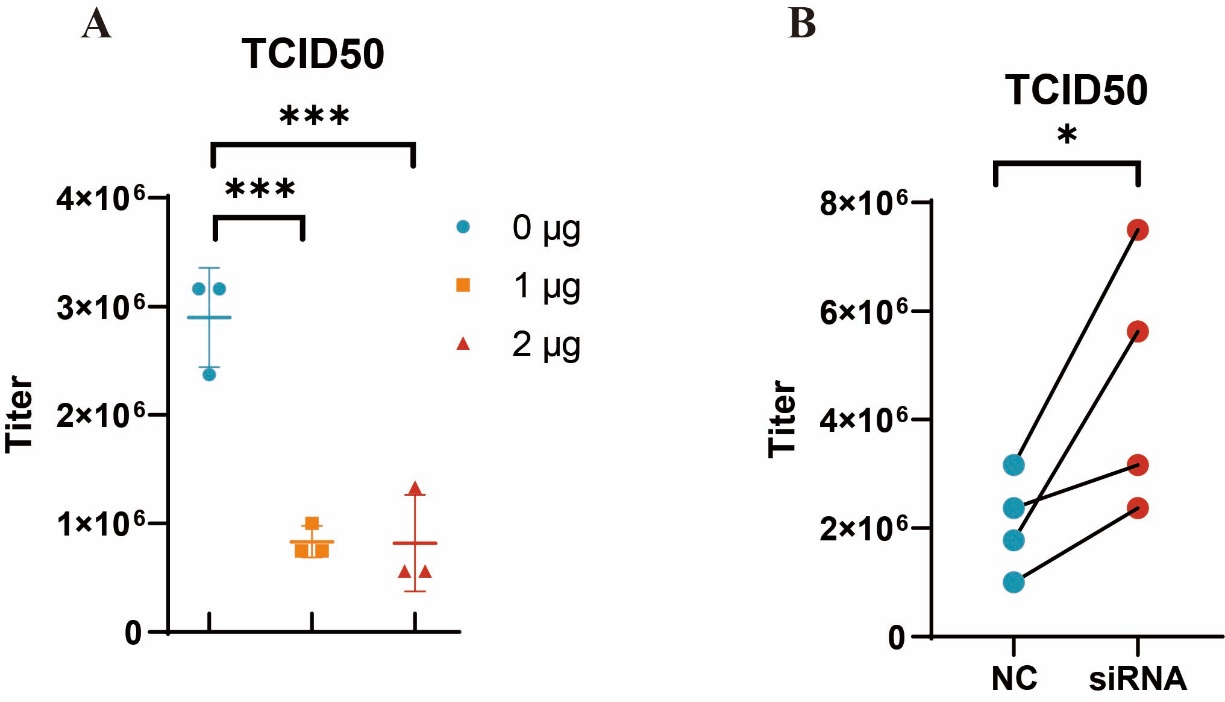


**Figure S6. GPR109A suppressed HSV replication.** (A) Raw 264.7 cells were transfected with 0, 1, 2 μg pcDNA3.1-GPR109A plasmid for 24 h then followed by HSV infection (MOI=1) for 12 h. The viral titers in the supernatants were measured by TCID50. pcDNA3.1 vector plasmid was used as control group and total DNA in each group is 2 μg. (B) Non-targeting and GPR109A-targeting siRNA were transfected in Raw 264.7 cells follow by HSV infection (MOI=1) for 12 h. The viral titers in the supernatants were measured by TCID50. Each point represents a biological replicate of the experiments. **P<* 0.01, ****P<* 0.001.

Figure S7.

**
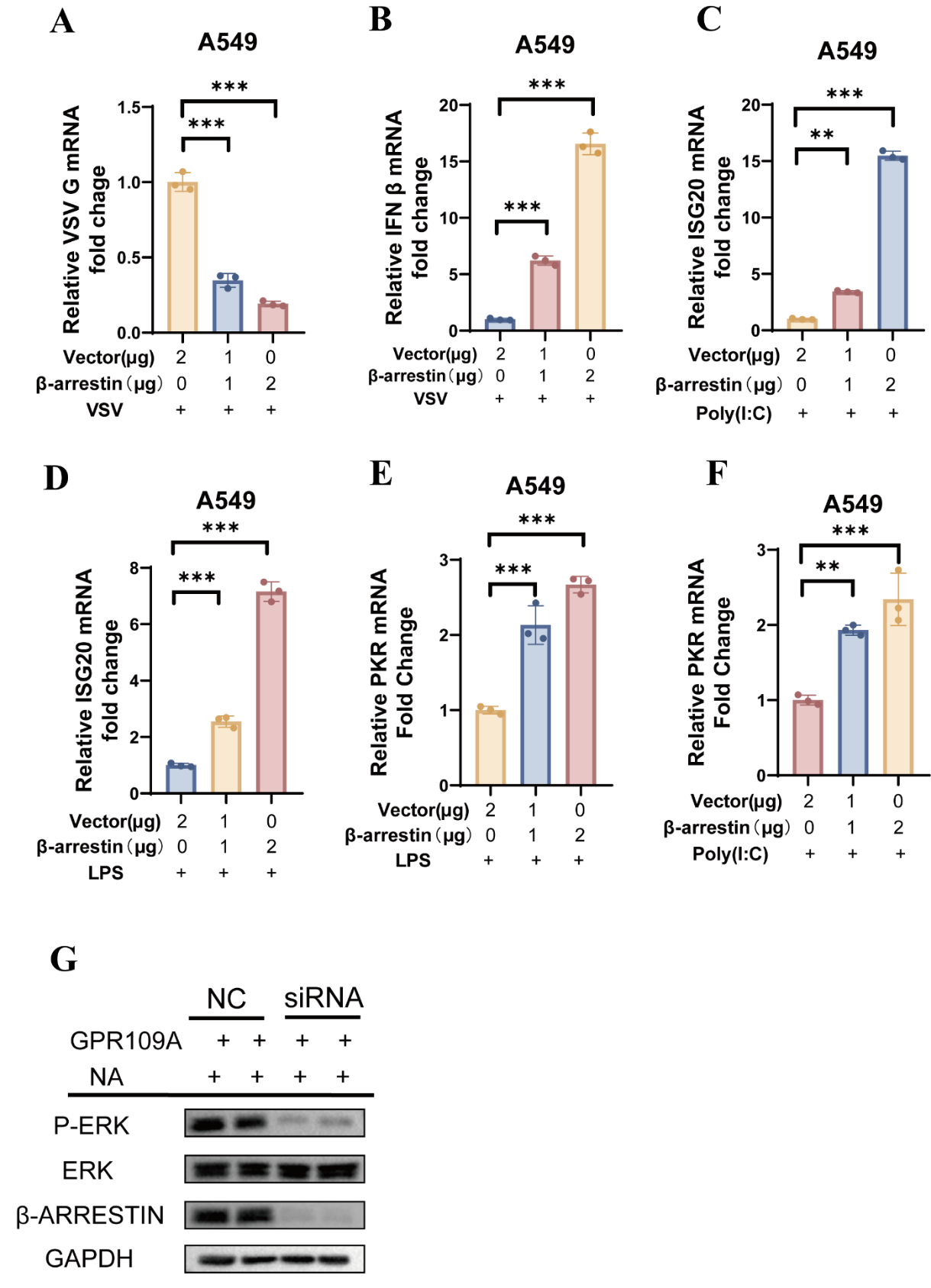
**

**Figure S7. Modulation of IFN signaling and ISGs expression by β-arrestin.** β-arrestin plasmid were transinfected in A549 cells for 24h. Then cells were incubated with VSV(MOI=0.1), 10μg/ml Poly(I:C) and 1 μg/ml LPS for 12h. Total RNA was extracted for RT-PCR analysis. The expression level of VSV-G protein (a) and IFN-β (B) was measured. (C-F) ISGs expression induced by Poly(I:C) and LPS was measured by RT-PCR. ***P<* 0.01, ****P<* 0.001.

Figure S8.


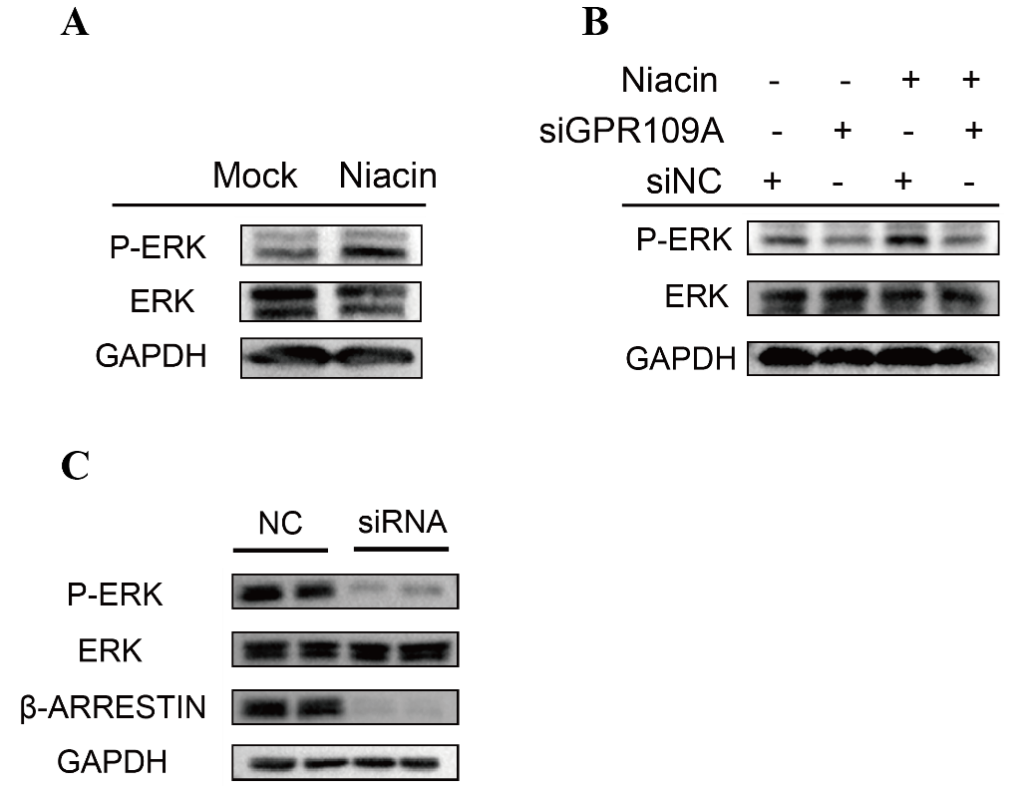


**Figure S8. Niacin promotes ERK phosphorylation through GPR109A and β-arrestin.** (A)300 μM niacin was incubated with A549 cells for 30 min and cell lysis were used for western blotting. (B) siRNA targeting GPR109A and control siRNA were transfected in A549 cells for 24h. Then 300 μM niacin was incubated with A549 cells for 30 min and cell lysis were used for western blotting. (C) siRNA targeting β-arrestin were transfected in A549 cells for 24h. Cells were incubated with 300 μM niacin for 30min and cell lysis were used for western blotting.

Figure S9.

**
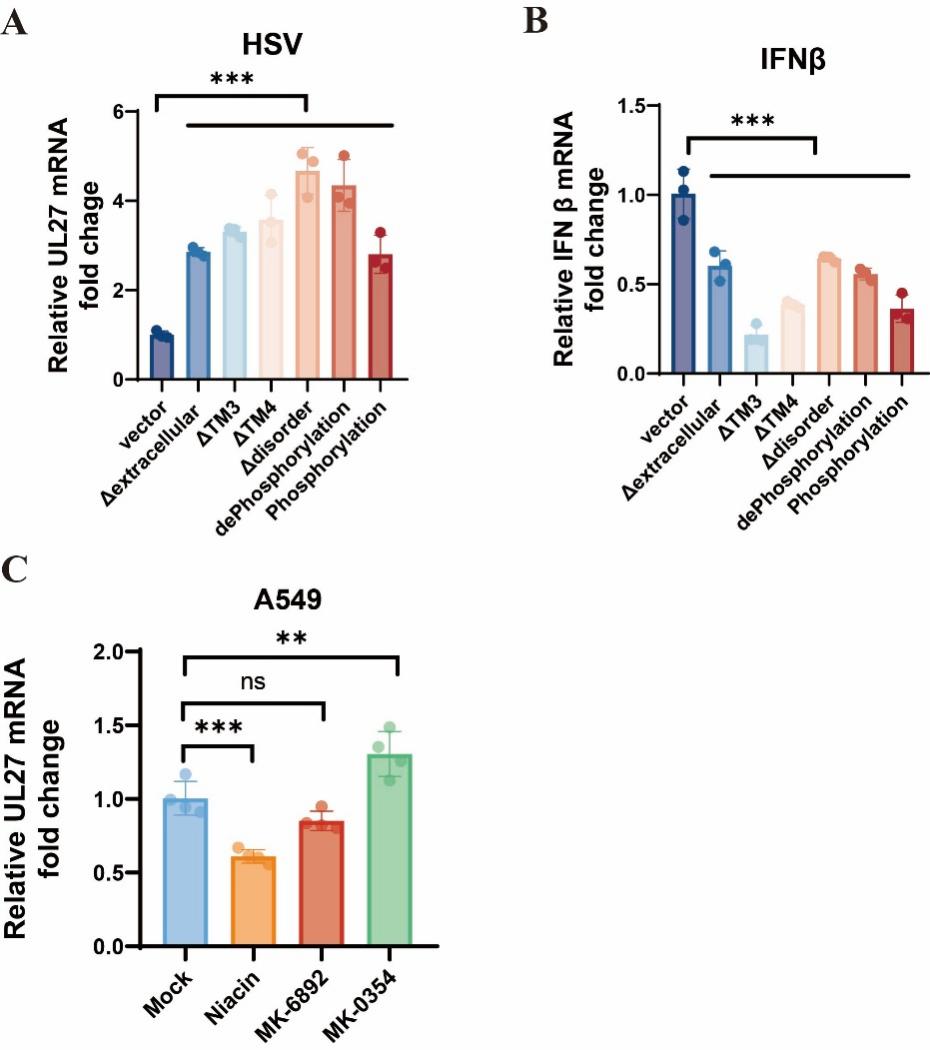
**

**Figure S9. The antiviral function of mutant and truncated GPR109A.**  Mutant and truncated GPR109A plasmids were transfected in A549 cells. After 24 h, cells were infected with HSV(MOI=0.1) for 12 h. and total RNA was extracted for RT-PCR analysis. The expression level of HSV UL27 (A) and IFN-β (B) was measured by RT-PCR analysis. Final data were presented as the mean ± SD of triplicate experiments. (C) A549 cells were treated with 1 μM niacin, 16 nM MK-6892, 1 μM MK-0354 and infected with HSV(MOI=0.1) for 12h. Total RNA was extracted for RT-PCR analysis. ***P<* 0.01, ****P<* 0.001.

Table S1. sgRNA sequence for GPR109 knockdown.

| Gene name | 5‘-3’ |
| --- | --- |
| *HCAR2* | CACCGGGACAACTATGTGAGGCGTTGTTT |

**Table S2. The sequences of primers for plasmid construction.**

| Plasmid | 5‘-3’ |
| --- | --- |
| pcDNA3.1-Flag-GPR109A | F: CCGGAATTCATGAATCGGCACCATCTGCA |
|  | R: TGGGCCCAACCTCTCCTTAATCTAGAGCA |
| pcDNA3.1-Flag-β-arrestin1 | F: CCGGAATTCATGGGCGACAAAGGGACGCGA |
|  | R: ACGTCTAGACTATCTGTTGTTGAGCTGTGG |
| pcDNA3.1-mus-GPR109A | F: TGCCTCGAGATGAGCAAGTCAGACCATTTTCTAG |
|  | R: ATGGGCCCTTAACGAGATGTGGAAGCCAGATAAG |

**Table S3. The sequences of siRNA used in this study.**

| Gene name | Sense（5’-3’） | antisense（5’-3’） |
| --- | --- | --- |
| *HCAR2*-Homo-326 | GGACAACUAUGUGAGGCGUTT | ACGCCUCACAUAGUUGUCCTT |
| *HCAR2*-Homo-386 | CUUCAUGUUGGCUAUGAACTT | GUUCAUAGCCAACAUGAAGTT |
| *HCAR2*-Homo-594 | GCAAAUUUGUGCAGCAGCUTT | AGCUGCUGCACAAAUUUGCTT |
| Mus*-HCAR2-*231 | CCACCUCAAGUCCUGGAAATT | UUUCCAGGACUUGAGGUGGTT |
| Mus*-HCAR2-*629 | GGUGGCACGAUGCUAUGUUTT | AACAUAGCAUCGUGCCACCTT |
| Mus*-HCAR2-*905 | CCCUUAGCUUUACCUACAUTT | AUGUAGGUAAAGCUAAGGGTT |
| Mus*-HCAR2-368* | GCCGUGUGAUGCUCUUCAUTT | AUGAAGAGCAUCACACGGCTT |
| *ARRB1*-homo-238 | GGACCCGAGUGUUCAAGAATT | UUCUUGAACACUCGGGUCCTT |
| *ARRB1*-homo-571 | CCUUUGAGAUCCCUCCAAATT | UUUGGAGGGAUCUCAAAGGTT |
| *ARRB1*-homo-667 | CCUUCUGCGCGGAGAAUUUTT | AAAUUCUCCGCGCAGAAGGTT |
| *ARRB1*-homo-818 | CCUAGAAGCCUCUCUGGAUTT | AUCCAGAGAGGCUUCUAGGTT |

**Table S4. The sequences of primers for RT-qPCR.**

| Primer name | 5‘-3 |
| --- | --- |
| Homo- IFNB1-F | GTCAGAGTGGAAATCCTAAG |
| Homo-IFNB1-R | ACAGCATCTGCTGGTTGAAG |
| Homo-IFNA1-F | GACTCCATCTTGGCTGTGA |
| Homo-IFNA1-R | TGATTTCTGCTCTGACAACCT |
| Homo-*ISG20*-F | TGGACTGCGAGATGGTGG |
| Homo-*ISG20*-R | GGGTTCTGTAATCGGTGAT |
| HSV-UL27-F | GCCTTCTTCGCCTTTCGC |
| HSV-UL-27-R | CGCTGTGCCCTTCTTCTT |
| Homo-*ARRB1*-F | CAAAGGGACGCGAGTGTTCA |
| Homo-*ARRB1*-R | GCAGGTCAGCGTCACATAGA |
| Homo-*HCAR2*-F | ACTATGTGAGGCGTTGGGAC |
| Homo-*HCAR2*-R | GGACCACCCGGAAATACCTG |
| Mus-*HCAR2*-F | AGGTGGCACGATGCTATGTT |
| Mus-*HCAR2*-R | ATGGCCCTCTTGATCTTGGC |
| Mus-β-actin-F | TGTTACCAACTGGGACGACA |
| Mus-β-actin-R | CTGGGTCATCTTTTCACGGT |
| Mus-IFN-β-F | TCACCTACAGGGCGGACTTC |
| Mus-IFN-β-R | TCTCTGCTCGGACCACCATC |
| Mus-TNF-α-F | GCCACCACGCTCTTCTGTCT |
| Mus-TNF-α-R | TGAGGGTCTGGGCCATAGAAC |
| Mus-IFNA4-F | TCAGGTCAAGGATAGTCTGGAG |
| Mus-IFNA4-R | AGGTTGTGTATTCCCACACTGTA |
| Homo-MX1-F | CTCCGACACGAGTTCCACAA |
| Homo-MX1-R | GGCTCTTCCAGTGCCTTGAT |
| VSV-G-F | TTGGCAAGTATGCTAAGTCAG |
| VSV-G-R | AGGACTTGAGATACTCACGAA |
| Homo-GAPDH-F | CAACAGCGACACCCACTCCT |
| Homo-GAPDH-R | CACCCTGTTGCTGTAGCCAAA |

**Table S5. The sequences of HSV-1 peptide used in this study.**

| **Name** | **Sequence (N’-C’)** |
| --- | --- |
| HSV-gB-1 | SSIEFARL |
| HSV-gB-2 | RMLGDVMAV |
| HSV-gB-4 | RFADIDTVIHA |
| HSV-gB-5 | EEYAYSHQL |
| HSV-gB-6 | RYMALVSAM |
| HSV-gB-7 | YYLANGGFL |
| HSV-gB-8 | APYKFKATM |
| HSV-gB-9 | VGHRRYFTF |
| HSV-gB-10 | REMIRYMALVSAME |
| HSV-gB-11 | EMIRYMALVSAMER |
| HSV-gD-1 | SLKMADPNRFRGKDLP |
| HSV-gD-2 | DPEDSALL |
| HSV-gD-3 | KYALADASLKMADPNRFRGKDLP |
| HSV-gD-4 | KYALVDASL |
| HSV-gD-5 | NYYDSFSAV |
| HSV-gD-6 | KAPYTSTLL |
| HSV-gD-7 | VAPQIPPNW |
| HSV-gD-8 | TPNATQPEL |
| HSV-gD-9 | CGIVYWMRRHTQKA |
| HSV-gD-10 | GIVYWMRRHTQKAPK |
| HSV-other-1 | QTFDFGRL |
| HSV-other-2 | GDEYDDAADAAGDRAP |
| HSV-other-3 | LGQPEEGAPCQVVLQ |
| HSV-other-4 | DYATLGVGV |

**Table S6. Information for antibodies used in this study.**

| Antibody | Source & Identifier | Dilution |
| --- | --- | --- |
| anti-GPR109A | Santa Cruz, sc-377292 | 1/1,000 |
| anti-STAT | Cell Signaling Technology, 19781S | 1/1,000 |
| anti-p-STAT1 | Cell Signaling Technology, 3504S | 1/1,000 |
| anti-ERK | Proteintech, 83533-1-RR | 1/1,000 |
| anti-phosphorylated ERK | Proteintech, 28733-1-AP | 1/1,000 |
| anti-GAPDH | Everest Biotech, EB06377 | 1/10,000 |
| anti-HSV-ICP0 | Santa Cruz, sc-53070 | 1/1,000 |
| anti-HSV-ICP27 | Santa Cruz, sc-69807 | 1/1,000 |
| Anti-VSV-g | Santa Cruz, sc-365019 | 1/1,000 |
| anti-FLAG | Sigma-Aldrich, F7425 | 1/3,000 |
| HRP-conjugated sheep anti-mouse IgG | Abcam, ab102458 | 1/10,000 |
| HRP-conjugated mouse anti-goat IgG | Bioss, bs-0294M-HRP-100ul | 1/10,000 |

Table S7. Information for compounds and other reagents used in this study.

| Compound to be screened | Source & Identifier |
| --- | --- |
| Acyclovir | Macklin, A829547 |
| Isonicotinic acid | Macklin, I811605 |
| Nicotinic acid | Macklin, N814566 |
| 1H-Pyrrole-2,3-dicarboxylic acid | Macklin, H900639 |
| 3,5-Dimethyl-1H-pyrrole-2,4-dicarboxylic acid | Macklin, D891202 |
| 6-Methyl-2,3-Pyridinedicarboxylic Acid | Macklin, M843164 |
| 3-Methylpyridine-2-carboxylic acid | Macklin, M825089 |
| 2-Methylnicotinic acid | Macklin, M835489 |
| Phthalic acid | Macklin, P815673 |
| 5-Ethylpyridine-2,3-dicarboxylic acid | Macklin, E835412 |
| 2-Picolinic acid | Macklin, P815506 |
| Other regents | Source & Identifier |
| Niacin | MCE, HY-B0143 |
| MK-6892 | MCE, HY-10680 |
| Poly(I:C) | GLPBIO, GC14710 |
| LPS | GLPBIO, GC19203 |
| PD98059 | MCE, HY-12028 |
